# Lung ultrasound training and evaluation for proficiency among physicians in a low-resource setting

**DOI:** 10.1186/s13089-021-00236-4

**Published:** 2021-06-30

**Authors:** Darlene R. House, Yogendra Amatya, Benjamin Nti, Frances M. Russell

**Affiliations:** 1 Department of General Practice and Emergency Medicine, Patan Academy of Health Sciences, Kathmandu, Nepal; 2 Department of Emergency Medicine, Indiana University School of Medicine, 720 Eskenazi Avenue, Indianapolis, IN 46202 USA

**Keywords:** Point-of-care ultrasound, Lung ultrasound, Proficiency, Education

## Abstract

**Background:**

Lung ultrasound (LUS) is helpful for the evaluation of patients with dyspnea in the emergency department (ED). However, it remains unclear how much training and how many LUS examinations are needed for ED physicians to obtain proficiency. The objective of this study was to determine the threshold number of LUS physicians need to perform to achieve proficiency for interpreting LUS on ED patients with dyspnea.

**Methods:**

A prospective study was performed at Patan Hospital in Nepal, evaluating proficiency of physicians novice to LUS. After eight hours of didactics and hands-on training, physicians independently performed and interpreted ultrasounds on patients presenting to the ED with dyspnea. An expert sonographer blinded to patient data and LUS interpretation reviewed images and provided an expert interpretation. Interobserver agreement was performed between the study physician and expert physician interpretation. Cumulative sum analysis was used to determine the number of scans required to attain an acceptable level of training.

**Results:**

Nineteen physicians were included in the study, submitting 330 LUS examinations with 3288 lung zones. Eighteen physicians (95%) reached proficiency. Physicians reached proficiency for interpreting LUS accurately when compared to an expert after 4.4 (SD 2.2) LUS studies for individual zone interpretation and 4.8 (SD 2.3) studies for overall interpretation, respectively.

**Conclusions:**

Following 1 day of training, the majority of physicians novice to LUS achieved proficiency with interpretation of lung ultrasound after less than five ultrasound examinations performed independently.

## Introduction

Lung ultrasound (LUS) is an effective tool to evaluate patients with dyspnea in the emergency department (ED) [1–5]. Guidelines recommend including LUS during point-of-care ultrasound training in emergency medicine [6]. As an operator-dependent skill, LUS requires adequate training in order to be used effectively [7]. In a recent systematic review of LUS training, Pietersen et al. recommends a three-step approach of teaching theoretical knowledge, followed by hands-on sessions on simulators or healthy subjects, and finally supervised scanning of patients to determine when learners are ready for independent scanning [8]. However, there is no standardized recommendation on the amount of training required or the number of examinations needed to achieve proficiency in LUS [8]. Guidelines for LUS competency are based on expert opinion and lack prospective data to support them [9].

Only a handful of prior studies have evaluated LUS proficiency in learners [10–15]. Several of these studies primarily focused on learners in the intensive care unit (ICU) with varied supervision and assessments of proficiency [10, 14, 15]. While Arbelot et al. included ED residents, the study was performed in the ICU setting [15]. Other studies primarily focused on particular disease processes, such as pneumothoraces [13] or patients with cardiogenic pulmonary edema [12]. There remains a lack of evidence regarding the amount of training and the number of LUS examinations needed to attain proficiency for evaluating the undifferentiated dyspneic patient presenting to the ED. This is of particular importance in a resource-limited setting where radiographs are harder to obtain. The objective of this study was to determine the threshold number of LUS examinations physicians need to perform to achieve proficiency for interpreting LUS in ED patients with dyspnea.

## Materials and methods

### Study design

This was a prospective study evaluating LUS training and proficiency of physicians performing lung ultrasound in the Patan Hospital Emergency Department. It was conducted from July 2017 through May 2019. This study was approved by the Nepal Health Research Council Ethical Review Board, and written consent was obtained from each physician participant.

### Study setting and population

Patan Hospital is a large urban teaching hospital affiliated with Patan Academy of Health Sciences. The ED has an annual patient volume of approximately 48,000 patients. The majority of care in the ED is provided by medical officers, physicians who have completed medical school and are preparing for post-graduate medical education. Patient care is supervised by faculty trained in either general practice or emergency medicine (EM).

All physicians working in Patan Hospital ED were eligible for inclusion in the study. Physician demographics, including medical position (i.e., medical officer, resident, fellow, faculty), and years of practice, were collected. A pre-training assessment of experience and confidence with ultrasound and specifically LUS was obtained.

Physicians performed LUS studies on patients presenting to the ED with dyspnea.

### Lung ultrasound training

A total of eight hours of training was provided for ED physicians that consented for participation in the study. The first four hours consisted of a 1-h didactic session on LUS followed by hands-on practice with a human model. This was then followed by four hours of one-on-one proctored scanning in the ED performing LUS on dyspneic patients to ensure learners were comfortable with acquiring images, saving images for submission, and interpreting images according to the BLUE protocol [2].

### Study protocol

Ultrasounds were performed using a SonoSite M-Turbo (Fujifilm SonoSite, Inc.) machine and a curvilinear probe. The ultrasound protocol included ten views of the lungs: two anterior, two lateral, and one posterior view on each hemithorax [2].

Following the training, each physician performed scans independently, recorded images in all zones labeled by location, and reported findings in each zone, including an overall interpretation based on the BLUE protocol [2, 16]. Physicians assessed for lung sliding, A-lines, B-lines, consolidations, and/or pleural effusions [2, 17]. A lines were defined as recurrent horizontal echogenic artifacts arising from the pleural line generated by sub-pleural air [2]. B-lines were defined as discrete vertical hyperechoic artifacts arising from the pleural line, extending to the bottom of the ultrasound screen, erasing A lines, and moving with lung sliding [2]. Consolidation was defined as sub-pleural hypoechoic or tissue-like area with B-lines at the far-field border [2].

Physician trainees recorded their interpretation of each zone and overall interpretation on a standardized data collection form immediately following each scan. These examinations were exported from the ultrasound machine and uploaded for review of quality and interpretation by one of two expert sonographers with registered diagnostic medical sonographer certification and > 1000 previously performed ultrasounds. Expert sonographers were blinded to clinical data and physician interpretations of each zone and overall interpretation. After every five scans, physicians received feedback on the quality and interpretation of their ultrasounds. Image quality was graded on a five-point scale (1—Very Poor, 2—Poor, 3—Average, 4—Good, 5—Excellent). Trainees were expected to submit a minimum of five abnormal scans, including both B-lines and consolidation, to ensure proficiency with both normal and abnormal findings [18].

### Statistical analysis

Cumulative sum (Cusum) statistical methodology was used to evaluate the number of LUS scans required to reach an adequate level of training [19, 20]. As described in more detail by Russell et al., Cusum analysis uses pre-defined acceptable and unacceptable failure rates and evaluates sequential data to determine when a learner has reached proficiency with a skill [12, 19, 20]. This statistical method assesses procedural competence for learners over time. Two outcomes were considered: whether physicians were at least 70% correct; and whether they had “Yes” for correct interpretation. This predetermined threshold has been used previously in the literature to determine learner competency [12, 21]. Assuming an accepted failure probability was 0.3 and smallest detectable failure probability was 0.7, type 1 error and type 2 error (alpha and beta, respectively) were both set at 0.1.

Interobserver agreement for ultrasound interpretations between the study physician and expert sonographer was calculated using Cohen’s Kappa coefficient. A random 10% of examinations was overread by a second expert sonographer to assess interobserver agreement between experts.

## Results

Twenty-one physicians were enrolled in the study; however, two physicians withdrew from the study after leaving the hospital for personal reasons, leaving nineteen physicians who completed the study. The majority of physicians in the study were medical officers (84%) who had less than 1 year clinical experience following medical school (Table 1). Most had never used an ultrasound machine or had used the ultrasound less than ten times. The general practice faculty and two emergency medicine fellows (general practice physicians doing an 18-month training in emergency medicine) had used the ultrasound a large amount, primarily performing obstetrical ultrasounds. None of the study physicians had performed LUS prior to the study.

**Table 1:** Demographics

Provider	No. of participants (*N* = 19)	Average experience post-medical school
Faculty	1	9 years
EM fellow	2	9 years
Medical officer	16	5 months (SD 2.5)

A total of 330 lung examinations were performed with 3288 lung zones included in analysis. Physicians submitted an average of 17 complete lung ultrasound scans (SD 5.5, range 10–30). Several examinations were missing posterior lung views due to patient’s clinical status and inability to sit up or roll over. One hundred and sixteen (35%) lung examinations were normal and 214 (65%) had abnormalities. The most common diagnosis for dyspnea in this ED population was pneumonia, one of the most common causes of morbidity and mortality in resource-limited settings (Table 2).

**Table 2:** Lung ultrasound diagnoses

Diagnosis	Number (*N* = 330)
Pneumonia	137
Pneumonia with effusion	50
COPD/asthma	86
Normal/viral illness	30
Pulmonary edema	19
Pleural effusion	4
Pneumothorax	3
Interstitial lung disease	1

**Fig. 1:**
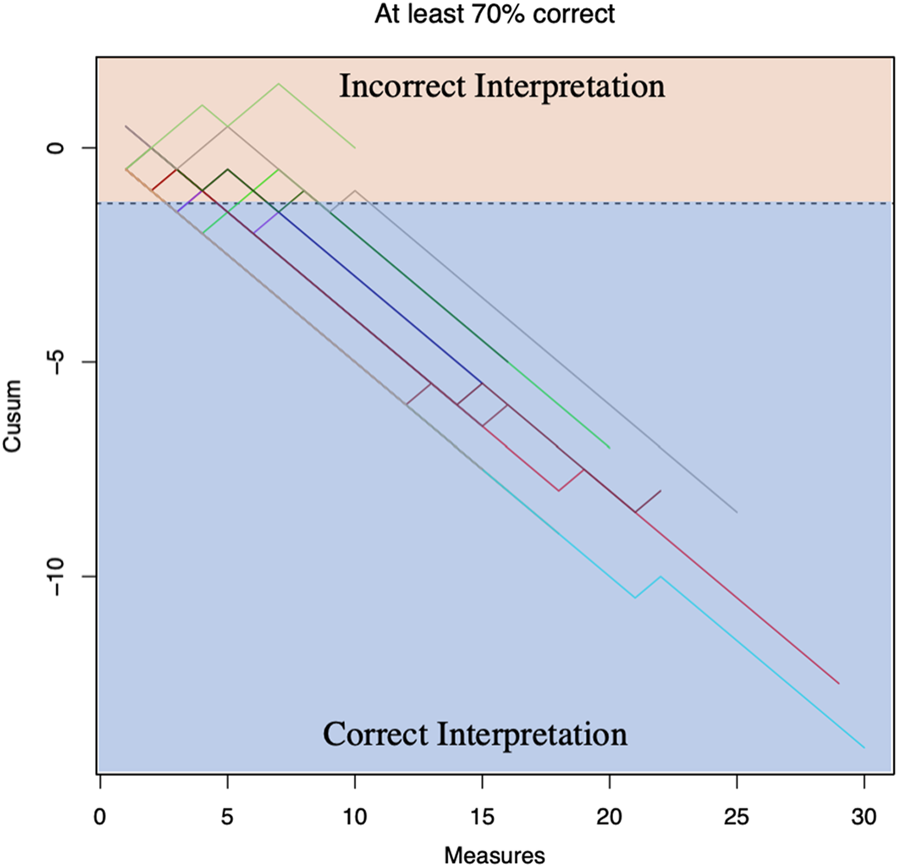
Cusum plot for number of scans required to reach proficiency

**Fig. 2:**
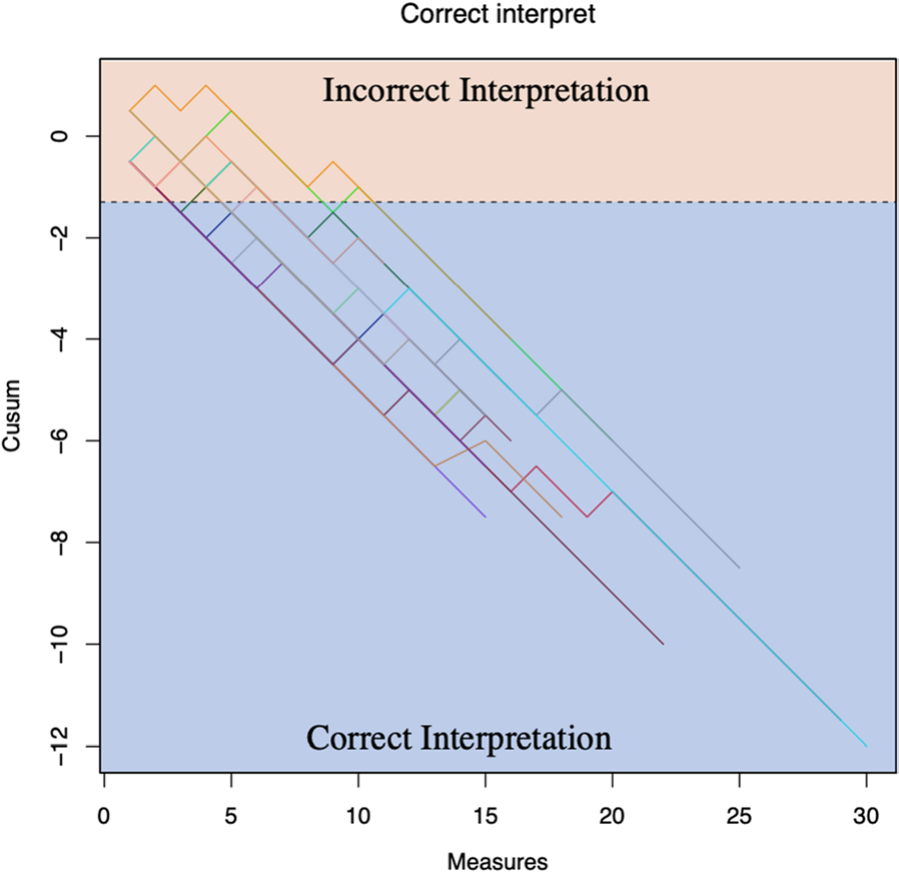
Cusum plot for number of scans required to reach a correct overall interpretation

Eighteen physicians (95%) reached proficiency. Physician S only provided 10 scans and did not reach proficiency in this analysis. We found that physicians attained proficiency for interpreting LUS accurately when compared to an expert after 4.4 (SD 2.2) LUS studies for individual zone interpretation and 4.8 (SD 2.3) LUS studies for overall interpretation; see Figs. 1, 2. Table 3 provides a summary of statistics for the number of scans required to reach an acceptable level of training by physician.

**Table 3:** Scans required for proficiency by physician

Physician	Number for proficiency with individual zone interpretation	Number for proficiency for overall interpretation
A	3	3
B	5	5
C	3	9
D	3	11
E	3	5
F	3	3
G	5	3
H	7	3
I	5	3
J	7	3
K	3	3
L	3	3
M	3	3
N	9	5
O	3	7
P	3	5
Q	3	7
R	3	5
S	> 10	5
Average (SD)	4.4 (2.2)	4.8 (2.3)

Physicians also demonstrated good image quality with an average image quality of 4.1 (SD 0.3). The image quality improved as more scans were performed.

Expert interobserver agreement for individual images was 0.7 and for overall diagnosis was 0.9.

## Discussion

This was the first study to evaluate the number of LUS scans needed to attain proficiency for acquiring and interpreting images on undifferentiated dyspneic ED patients in a resource-limited setting. Overall, we found that nearly all novice users became proficient in acquiring and interpreting lung ultrasounds after performing on average less than five independent examinations when compared to an expert.

These results are similar to the study by See et al., evaluating proficiency in lung ultrasound use by respiratory therapists in an intensive care setting [10]. In their study, respiratory therapists only needed ten directly observed scans to accurately obtain and interpret images. However, these exams were all directly observed and may provide an added confidence that may not be there when scanning independently. This study did not follow learners after supervised scans to evaluate if proficiency was maintained while scanning independently. In our study, novice physicians scanned independently and were able to attain proficiency within the same number of scans.

Millington et al. developed and evaluated a tool for assessment of competency in thoracic ultrasonography on ten learners, finding learners rapidly improved in image generation and interpretation in up to 25–30 scans [11]. However, the study did not evaluate sequential ultrasounds from learners or define when competency was achieved. Similarly, a training curriculum and prospective evaluation by Arbelot et al. recommended 20–25 supervised scans to acquire basic skills for interpretation of LUS. [15] While our study provided five supervised scans, we found proficiency was obtained after only an additional five scans performed independently. This difference may be due to differences in the complexity of ICU versus ED patients, which highlights the need for further evaluation of proficiency needs for learners in different settings.

Russell et al. found that novice sonographers, including physicians and non-physicians, achieved proficiency in LUS B-line quantification after limited training and independently scanning 11 lung zones (less than two full LUS examinations) [12]. This study assessed 29 learners and used Cusum analysis to construct learning curves [12]. It differs from our study in that they specifically evaluated proficiency for B-line quantification in acute heart failure patients instead of determining LUS findings and diagnosis in ED patients with undifferentiated dyspnea. The data from both of these studies remain consistent with prior research suggesting that LUS is one of the easier ultrasound exam types to perform and interpret [22].

In our study, one learner did not reach proficiency. Soon after enrollment into the study, the learner transitioned from the ED to a clinical research position. This transition may have resulted in less vested interest in learning ultrasound. Additionally, the learner tried to quickly complete the number of ultrasounds, which may have made it difficult to implement any feedback to develop proficiency. While all other learners obtained proficiency within ten scans, this highlights the importance that some learners may require more scans.

There are several limitations within this study. We had a small sample size and conducted the study within one center, making generalizability difficult. Also, there may be a selection bias as physicians volunteered to participate in the study, which may lead to those physicians with more motivation to learn ultrasound being included in the study. Additionally, we used the BLUE protocol for interpretation of LUS findings. The BLUE protocol was developed in the ICU setting on critically ill patients, and does not differentiate subtle findings (i.e., interstitial infections from edema or pneumonia from other causes of consolidation). However, the BLUE protocol provides a helpful algorithm for novice users in learning how to apply lung ultrasound to clinical care. Because of this, we utilized this organized approach in our training and encouraged learners to interpret these findings within the clinical context. We did not collect body mass index (BMI) or other patient demographics for the study as this is difficult to do in a resource-limited setting. BMI may impact image quality and thus interpretation. Although lung ultrasound is considered easy to acquire [22], future studies should assess impact of BMI on proficiency. Finally, interpretation is dependent on clinical context and we did not follow patients to ensure that the clinical diagnosis matched the LUS interpretation. It is important to recognize that with LUS it is relatively easy to learn how to acquire images; however, interpretation of artifacts has a steeper learning curve. In this study, we found that learners achieved proficiency for interpretation after just five independent scans. When applying LUS clinically, it is important to remember that findings and subsequent management of the patient should be done within the clinical context.

Overall, this study further helps to understand LUS proficiency and may be used to help guide future benchmark recommendations. Having a better understanding of the number of scans necessary to reach proficiency is crucial to determine the level of training needed for providers using LUS. Future studies evaluating ongoing proficiency and impact on clinical care would be helpful, particularly in resource-limited settings where other diagnostic tools are lacking.

## Conclusion

Following 8 h of didactic and hands-on training, the majority of physicians novice to lung ultrasound achieved proficiency with interpretation of lung ultrasound after less than five ultrasound examinations performed independently.

## Data Availability

Not applicable.

## Abbreviations

LUS: Lung ultrasound
ED: Emergency department
ICU: Intensive care unit
EM: Emergency medicine
Cusum: Cumulative sum
BMI: Body mass index
